# Urinary Analysis of Fluid Retention in the General Population: A Cross-Sectional Study

**DOI:** 10.1371/journal.pone.0164152

**Published:** 2016-10-20

**Authors:** Robert G. Hahn, Nina Grankvist, Camilla Krizhanovskii

**Affiliations:** Research Unit, Södertälje Hospital, 152 86, Södertälje, Sweden; The University of Manchester, UNITED KINGDOM

## Abstract

**Objective:**

Renal conservation (retention) of fluid might affect the outcome of hospital care and can be indicated by increased urinary concentrations of metabolic waste products. We obtained a reference material for further studies by exploring the prevalence of fluid retention in a healthy population.

**Methods:**

Spot urine sampling was performed in 300 healthy hospital workers. A previously validated algorithm summarized the urine-specific gravity, osmolality, creatinine, and color to a fluid retention index (FRI), where 4.0 is the cut-off for fluid retention consistent with dehydration. In 50 of the volunteers, we also studied the relationships between FRI, plasma osmolality, and water-retaining hormones.

**Results:**

The cut-off for fluid retention (FRI ≥ 4.0) was reached by 38% of the population. No correlation was found between the FRI and the time of the day of urine sample collection, and the FRI was only marginally correlated with the time period spent without fluid intake. Volunteers with fluid retention were younger, generally men, and more often had albuminuria (88% vs. 34%, *P* < 0.001). Plasma osmolality and plasma sodium were somewhat higher in those with a high FRI (mean 294.8 vs. 293.4 mosmol/kg and 140.3 vs. 139.9 mmol/l). Plasma vasopressin was consistently below the limit of detection, and the plasma cortisol, aldosterone, and renin concentrations were similar in subjects with a high or low FRI. The very highest FRI values (≥ 5.0, N = 61) were always accompanied by albuminuria.

**Conclusion:**

Fluid retention consistent with moderate dehydration is common in healthy staff working in a Swedish hospital.

## Introduction

Moderate dehydration is a difficult condition to detect in clinical medicine. One method developed in sports medicine is based on the concept that high urinary concentrations of metabolic waste products indicate dehydration. The loss of body water caused by physical activity causes the kidneys to conserve (retain) water, thereby raising the specific gravity of the urine, increasing the osmolality and the creatinine concentration, and causing darkening of the urine color [1–3]. This concentration process can be prevented by appropriate replacement of the body water lost during exercise. The medical importance of chronic moderate dehydration has not been thoroughly investigated, whereas severe dehydration has undisputed effects on health by reducing physical and intellectual performance [3–5].

Urine analysis could also be potentially useful in hospital patients. Recent studies indicate that fluid retention is relevant to hospital care [6–8] and even impairs health-related outcomes [9–11]. However, one third of 57 healthy subjects had high urinary concentrations of waste products even *before* performing recreational sports [12]. Moreover, concentrated urine was found in 36% of on-call nurses and doctors [13]. These observations suggest that water conservation could be a common trait in the general population.

The aim of the present observational study was to examine in greater detail the frequency of fluid retention in the general population and to search for a possible hormonal reason. These data would be a useful reference for assessment of fluid retention in hospital patients. The hypothesis was that fluid retention consistent with moderate dehydration is as common in the general population as was previously found in clinical patients. For this purpose, urinary analysis was performed on 300 hospital workers. In addition, 50 of these workers provided blood samples for analysis of plasma osmolality and the concentrations of water-retaining hormones.

## Materials and Methods

During three weeks in October 2014, 300 hospital workers were enrolled in the present cross-sectional study. The study was conducted according to the guidelines of the Declaration of Helsinki and the Regional Ethics Committee of Stockholm (June 12, 2013, Dnr. 2013/903-31/1, Chairperson Olof Forssberg) specifically approved this study. Written consent was obtained from all participants. The study was advertised on the local Internet system as an invitation to all healthy staff, and enrolment was stopped when the planned number of participants had been reached. In a few cases, close relatives were invited in order to include subjects of more advanced age.

### Procedure

The volunteers were asked to provide two fresh 10 ml spot urine samples at the Research Unit during a normal working day. They were instructed not to ingest any fluid within two hours prior to the sampling to allow the urine to reflect the steady-state situation. Fifty of the volunteers also agreed to provide blood samples for hormonal analysis at the same time. The purpose of the blood analyses was to identify possible correlations between renal water conservation, as evidenced by the urine analysis, and plasma osmolality and a number of water-retaining hormones that act on the kidneys.

All participants completed an anonymous questionnaire that collected information about gender, age, height, body weight, time of last fluid intake, time when the urine sample was taken, existing diseases, regular medications (if any), and time of last menstruation (if present). Participation was prohibited during ongoing menstruation. The questionnaire could be tracked to the results of the urine and blood analyses, but could not be linked to any individual volunteer.

### Measurements

The urine color was graded immediately by comparing the samples to a urine color chart (available at www.hydrationcheck.com) [1]. All samples were analyzed for concentrations of sodium, potassium, and osmolality at the certified clinical chemistry laboratory at Karolinska University Hospital in Solna, Stockholm, Sweden, on the same day as the urine sample was delivered.

The albumin and creatinine concentrations and the albumin/creatinine ratio were also quantitatively measured on the fresh samples using bedside equipment (DCA Vantage Analyzer, Siemens Healthcare Diagnostic, Erlangen, Germany).

The urine specific gravity, pH, and the urinary glucose, erythrocyte, protein, urobilinogen, nitrite, and leucocyte contents were also measured on fresh samples using a Clinitek Status^®^ Analyzer (Siemens Healthcare Diagnostics). The urine specific gravity is expressed in steps of 0.005 (no unit) based on changes in pK_a_ for pre-treated poly-electrolytes.

Blood samples were centrifuged at room temperature and sent to the Karolinska University Hospital for analysis (within 24 hours) of osmolality and the serum concentrations of sodium, potassium, calcium, and cortisol. The plasma concentrations of renin and aldosterone were measured in blood samples collected in EDTA tubes and centrifuged at 4°C. Blood samples collected in ice-cold sodium-heparin tubes were used to measure the fasting plasma concentration of vasopressin. These samples were immediately placed in an ice-box and centrifuged at 4°C within 30 min of the blood collection. All samples were handled in accordance with the instructions issued by the certified clinical chemistry laboratory at Karolinska University Hospital.

### Calculation of the Fluid Retention Index (FRI)

Analysis of the urine for metabolic waste products that appear in higher concentrations when the kidneys conserve fluid has previously been based on urine color, specific gravity, osmolality, and creatinine level. Urine color reflects the breakdown of erythrocytes. Specific gravity and osmolality represent the weight and the number of dissolved molecules (ions and other solutes) in the urine. Creatinine is an end-product of muscle metabolism.

Measuring a single one of these biomarkers would be sufficient for the detection of fluid retention in healthy subjects with a well-controlled diet. However, in hospital patients, each of these biomarkers could be altered by diet, disease, or medication. This problem is overcome by weighting these four markers together to create a composite value called the *Fluid Retention Index (FRI)*, which is based on ranges of agreement between the four biomarkers [12]. Each value of a marker is assigned a score, where a higher value indicates stronger fluid retention (Table 1). The mean of these four scores constitutes the FRI value, which then offers a more robust measure of fluid retention than is obtained using any one of its four components.

**Table 1:** Scheme for calculating the fluid retention index (FRI), which is the mean of the FR scores for four urinary indexes of fluid retention.

Fluid retention **score**	1	2	3	4	5	6
Specific gravity	≤ 1.005	1.010	1.015	1.020	1.025	1.030
Osmolality (mosmol/kg)	< 250	250–450	450–600	600–800	800–1000	> 1000
Creatinine (mmol/L)	< 4	4–7	7–12	12–17	17–25	> 25
Color* (shade)	1	2	3	4	5	6

* One score higher was used in the study due to incongruence with the other markers.

The composition of the index is then checked for outliers, which are determined by calculating the standard deviation (SD) for the mean value of the four scores. An outlier typically raises the SD to > 1.0. The individual scores are then reviewed and any single outlier is omitted, followed by recalculation of the index. The new value is accepted if SD ≤ 1.0, whereas the index is discarded as a failure if the SD still exceeds 1.0 [10].

The criterion for fluid retention was a FRI score ≥ 4.0, which corresponds to the degree of renal conservation of fluid that accompanies dehydration amounting to 3% of the body weight (specific gravity ≥ 1.020, creatinine ≥ 12 mmol/L, color ≥ 4, and osmolality ≥ 600 mosmol/kg) [12]. A specific gravity of ≥ 1.020 is also a frequently recommended threshold for dehydration in sports medicine [14].

To support that FRI reflects fluid retention, a screening analysis of the urinary concentrations of 36 metals was performed using atomic fluorescence spectroscopy (ICP-SMS method, performed by ALS Scandinavia AB, Luleå, Sweden) [15] in a subgroup of 15 samples with FRI values in the low range and 18 with FRI values in the high range.

The possible association of fluid retention with shedding of the endothelial glycocalyx layer [16] was assessed by measuring the urinary concentrations of syndecan-1 and hyaluronic acid in a random selection of 46 volunteers having FRI in the low range and 51 in the high range using commercially available ELISA kits (Human sCD138/Syndecan-1, Diaclone, France, and Hyaluronan Immunoassay, R&D Systems, Inc., MN).

### Statistics

The raw data is available as S1 Table. Normally distributed data were presented as the mean and (SD) and skewed distributions as the median (25th-75th percentiles). Differences between groups were evaluated by analysis of variance (ANOVA) or Mann-Whitney´s U test, as appropriate, and differences in frequencies by contingency table analysis. Correlations between parameters were studied by simple and multiple linear regression analysis. The statistical software was StatView SE+Graphics (Abacus Concepts, NJ). *P* < 0.05 was considered statistically significant.

## Results

### Quality control

**Fig 1:**
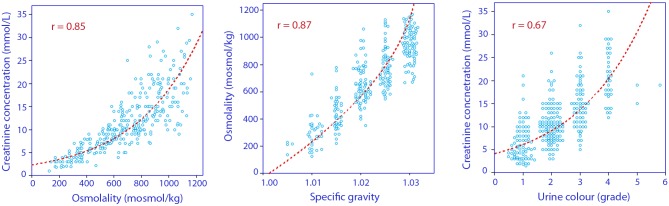
Inter-correlations between the four urinary markers used to calculate the fluid retention index (FRI). Overlapping points may have been separated for clarity.

Statistically significant non-linear correlations were found between the four urinary markers of fluid retention (Fig 1). The data were graded as shown in Table 1.

Quality control evaluation showed that the mean of the four markers had a SD that exceeded 1.0 in 59 of the 300 urine samples (20%). Upon removal of an outlier, which was most frequently the urine color, recalculation showed that SD dropped to ≤1.0 in 37 of these volunteers, who were included in further analyses. However, 22 samples (7.3%) were excluded from further analysis because the SD still remained > 1.0.

### Fluid retention index (FRI)

**Fig 2:**
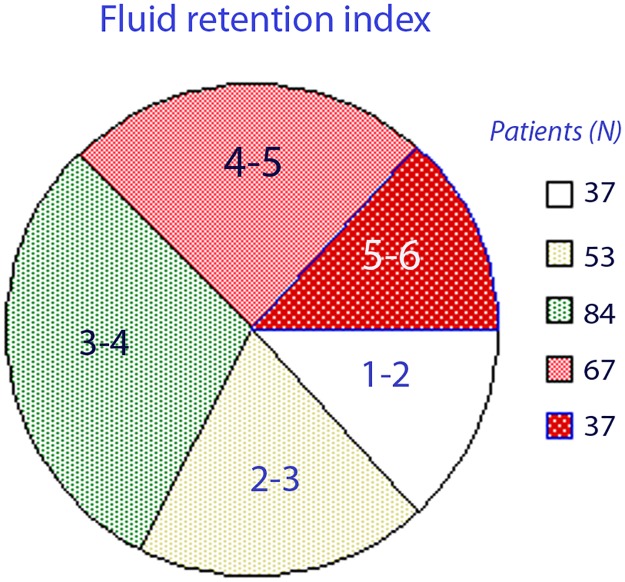
Distribution of volunteers between ranges of fluid retention index (FRI). Values of 4 and higher are considered to indicate fluid retention consistent with dehydration.

The overall FRI score was 3.7 (1.2) when based on the remaining 278 urine samples. Their distribution is shown in Fig 2.

**Fig 3:**
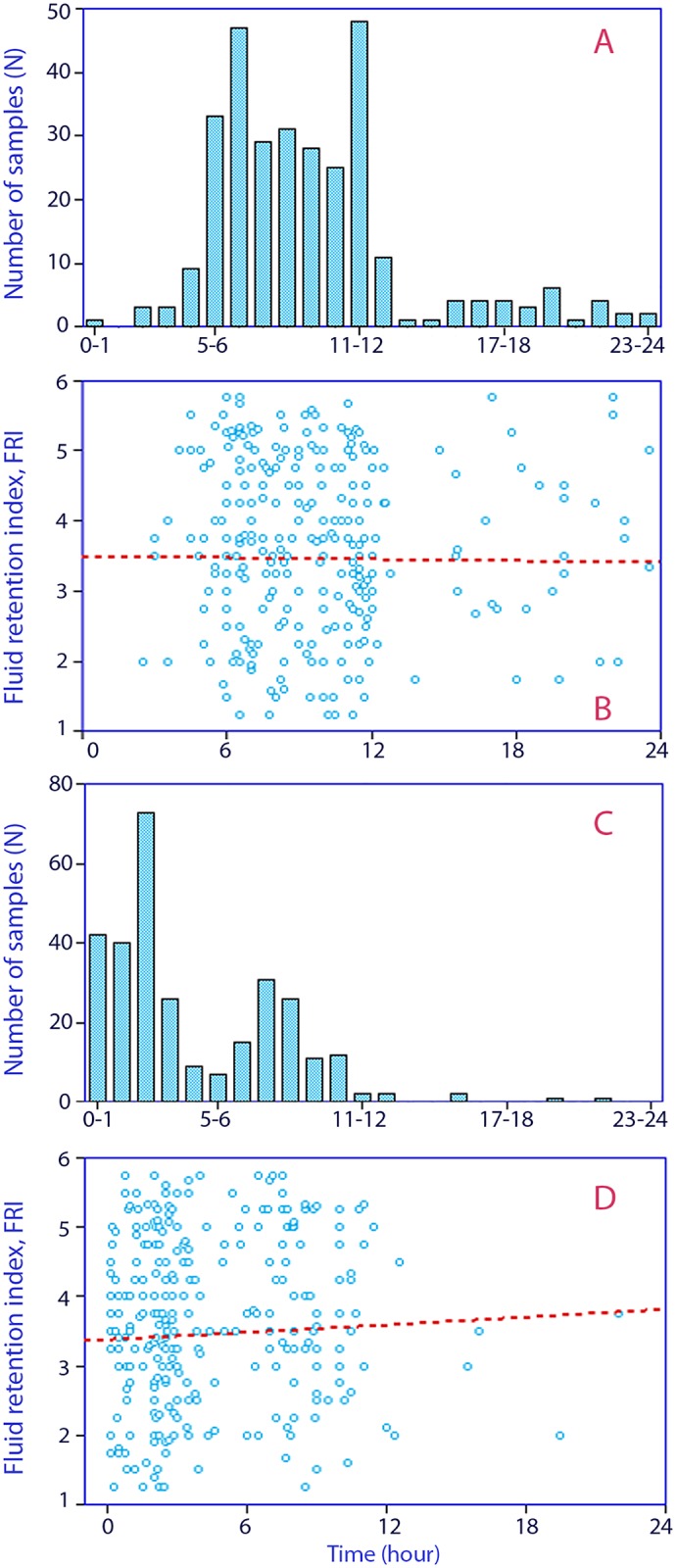
(**A**) Hour of the day when the urine sample was taken. 0 = midnight. (**B**) Fluid retention index depending on the time of day when urine sample was taken. (**C**) Period of time during which volunteers abstained from fluid intake before voiding. (**D**) Fluid retention index depending on the time period without fluid intake.

Most samples were taken between 6 AM and 2 PM (Fig 3A), but no correlation was evident between FRI and the time of the day when the urine was sampled (Fig 3B).

The volunteers had most often abstained from fluid intake for three hours (Fig 3C). No significant correlation was noted between FRI and the time between urine sampling and the last fluid intake (Fig 3D).

A total of 105 five volunteers (37%) had an FRI of ≥ 4.0, which is a previously used cut-off for fluid retention consistent with dehydration [11]. Differences between these volunteers and those with a lower FRI values are displayed in Table 2.

**Table 2:** Differences in measured parameters between volunteers who had a fluid retention index (FRI) consistent with dehydration (≥ 4.0) and those who did not fulfil this criterion. Data are the mean (SD).

	FRI < 4.0 (*N* = 158)	FRI ≥ 4.0 (*N* = 120)	Statistics
Demographics			
N	169	109	
Males, (N, %)	37 (22%)	42 (38%)	P< 0.01
Age (years)	45 (13)	41 (14)	P< 0.02
Height (cm)	168 (9)	171 (9)	P< 0.01
Weight (kg)	72 (16)	76 (13)	P< 0.02
Sampling (hour of the day)	9.6 (3.8)	9.7 (4.3	NS
Time without liquid (h)	4.6 (4.0)	4.6 (3.3)	NS
Urine analyses			
FRI (arbitrary unit) **	2.9 (0.8)	4.9 (0.5)	P<0.001
Specific gravity (no unit)	1.017 (0.005)	1.027 (0.003)	P<0.001
Osmolality (mosmol/kg)	508 (186)	897 (138)	P<0.001
Creatinine (mmol/l)	7.2 (3.1)	17.3 (5.6)	P<0.001
Color (shade)	2.6 (0.6)	4.0 (1.0)	P<0.001
Sodium (mmol/L)	92 (45)	127 (47)	P< 0.001
Potassium (mmol/L)	48 (29)	72 (29)	P< 0.001
Erythrocytes, trace (N, %)	13 (8%)	11 (10%)	NS*
pH	6.2 (0.7)	5.7 (0.5)	P< 0.001
Urobilinogen	3.5 (2.6)	3.3 (1.1)	NS
Nitrite positive	2 (1%)	2 (2%)	NS*
Leucocytes (N, %)	37 (22%)	7 (6%)	P< 0.001*
Albuminuria (N, %)	58 (34%)	96 (88%)	P< 0.001*
Syndecan-1 (ng/ml)	210 (172)	204 (161)	NS
Hyaluronic acid (ng/ml)	10.6 (4.7)	21.2 (7.0)	P<0.001
Blood analyses			
Osmolality (mosmol/kg)	293.4 (3.9)	294.9 (3.9)	NS
Sodium (mmol/L)	139.9 (1.6)	140.3 (2.1)	NS
Potassium (mmol/L)	4.2 (0.3)	4.1 (0.3)	NS
Calcium (mmol/L)	2.39 (0.07)	2.39 (0.08)	NS
Cortisol	404 (167)	347 (115)	NS
Aldosterone	334 (192)	304 (190)	NS
Renin	21 (12)	26 (14)	NS
Vasopressin (pg/ml)	< 1.5	< 1.5	NS

One-way ANOVA was used for statistics except * contingency table analysis.

** grouping variable.

NS = not significant

The urinary concentration of hyaluronic acid, but not of syndecan-1, increased with the FRI value (Table 2). Volunteers with a high FRI also had significantly higher urinary concentrations of 32 of the 36 metals tested, when compared with those with a low FRI (Table 3).

**Table 3:** Urinary concentrations of metals depending on the presence of water retention (FRI ≥ 4.0). Data are the median and 25th-75th percentiles.

Metal analysis	FRI < 4.0 (n = 15)	FRI ≥ 4.0 (n = 18)	Statistics*
Aluminum (μg/l)	3.0 (2.2–4.2)	7.5 (6.0–9.0)	P< 0.001
Antimony (μg/l)	0.02 (0.02–0.05)	0.10 (0.07–0.13)	P< 0.001
Barium (μg/l)	0.80 (0.65–1.13)	2.54 (1.66–5.4)	P< 0.001
Boron (mg/l)	1.42 (1.10–2.14)	3.06 (2.08–4.15)	P< 0.002
Bromine (mg/l)	1.68 (1.26–2.88)	4.36 (3.29–6.36)	P< 0.001
Cadmium (μg/l)	0.14 (0.09–0.20)	0.51 (0.38–0.95)	P< 0.001
Calcium (mg/l)	41 (27–61)	154 (92–182)	P< 0.001
Cesium (μg/l)	2.5 (1.8–3.9)	10.7 (8.1–14.9)	P< 0.001
Cobalt (μg/l)	0.08 (0.05–0.18)	0.40 (0.25–0.99)	P< 0.001
Copper (μg/l)	3.8 (3.0–4.4)	16.3 (15.0–20.1)	P< 0.001
Iodine (μg/l)	304 (198–373)	430 (344–593)	P< 0.02
Lead (μg/l)	0.33 (0.22–0.49)	0.86 (0.78–1.09)	P< 0.001
Lithium (μg/l)	5.96 (3.42–6.66)	23.5 (15.3–33.9)	P< 0.001
Magnesium (mg/l)	25.5 (20.3–29.9)	110.0 (84.3–120.0)	P< 0.001
Manganese (μg/l)	0.25 (0.16–0.60)	0.36 (0.14–1.00)	P< 0.005
Mercury (μg/l)	0.13 (0.10–0.29)	0.44 (0.36–0.75)	P< 0.001
Molybdenum (μg/l)	9.0 (5.8–19.0)	79.1 (50–130)	P< 0.010
Nickel (μg/l)	0.50 (0.50–0.93)	2.24 (0.50–3.29)	P< 0.003
Palladium (μg/l)	0.02 (0.02–0.04)	0.06 (0.05–0.08)	P< 0.001
Phosphorous (g/l)	0.17 (0.11–0.26)	1.20 (0.81–1.50)	P< 0.001
Potassium (g/l)	0.85 (0.62–1.2)	2.8 (1.8–3.6)	P< 0.001
Rhenium (μg/l)	0.01 (0.01–0.02)	0.04 (0.03–0.06)	P< 0.001
Rubidium (mg/l)	0.62 (0.53–0.92)	3.0 (2.0–3.1)	P< 0.001
Selenium (μg/l)	6 (5–10)	39 (30–60)	P< 0.001
Silicon (mg/l)	2.4 (2.0–3.4)	11.0 (7.8–15.8)	P< 0.001
Sodium (g/l)	0.88 (0.69–1.01)	2.5 (2.0–3.5)	P< 0.001
Strontium (μg/l)	33 (23–43)	146 (105–180)	P< 0.001
Sulphur (mg/l)	170 (152–239)	920 (780–1,100)	P< 0.001
Thallium (μg/l)	0.09 (0.08–0.15)	0.35 (0.30–0.45)	P< 0.001
Titanium (μg/l)	0.94 (0.63–1.56)	3.93 (2.71–4.61)	P< 0.001
Tungsten (μg/l)	0.03 (0.02–0.04)	0.11 (0.09–0.34)	P< 0.001
Zinc (μg/l)	105 (61–140)	720 (391–933)	P< 0.001
FRI (arbitrary unit)	2.00 (1.53–2.19)	5.25 (5.25–5.75)	P< 0.001**

* Mann-Whitney´s U test was used for statistics.

** Grouping variable.

### Gender

Seventy-one percent of the volunteers were women. They had a lower FRI values compared to the men, at 3.5 (1.2) *versus* 4.1 (1.2) (*P* < 0.001). This difference remained when the time the urine was sampled and the time period without intake of liquid were included in a multiple regression analysis. A detailed comparison of the urine and blood analyses depending on gender is given as S2 Table.

In total, 65 of the 199 women with an FRI value that passed the quality control still had menstruations, although not at the time of the urine sampling. During the first 5 days of the menstruation cycle, the FRI was 3.7 (1.6), at days 6–14 it scored 3.5 (1.1), and from day 14 and onward, including 4 women with gestagen-containing intrauterine coil, the FRI was 3.7 (1.3). The FRI in the women who no longer had menstruations was 3.5 (1.1). These differences were not statistically significant.

### Albuminuria

**Fig 4:**
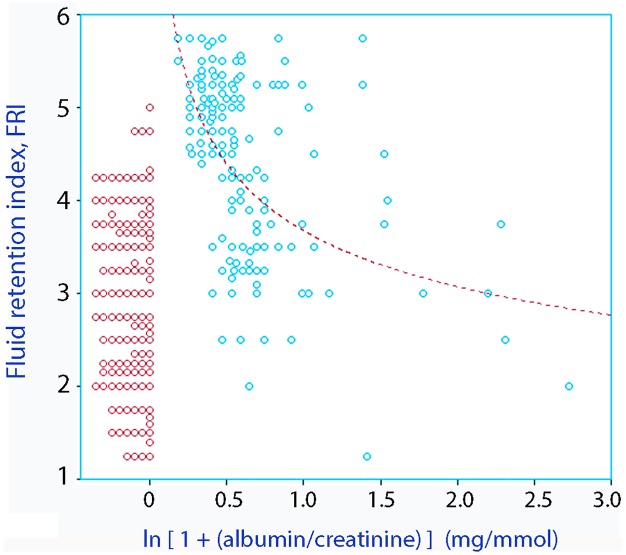
(A) Relationship between the degree of albuminuria and the fluid retention index. Points at zero and below denote absence of albuminuria. Overlapping points may have been separated for clarity. Albuminuria is displayed as ln (1 + albumin/creatinine) to highlight the values close to zero. On this scale, micro-albuminuria corresponds to 0.667.

Albuminuria was detected in 154 volunteers (54%). Albuminuria and FRI showed a biphasic relationship (Fig 4). Only 12 volunteers fulfilled the criterion of micro-albuminuria (> 2.5 albumin mg/mmol of creatinine) [17,18].

### Multivariate analysis

Stepwise multiple regression analysis showed that FRI was significantly, and independently, associated with albuminuria (presence: +), gender (male: +), age (-), and time without liquid (+). The *F* to remove the four factors was 163, 16, 7, and 5. The final correlation coefficient was 0.66.

## Discussion

Renal fluid conservation (retention) was common in our cohort of healthy hospital workers. As many as 2 out of 5 of the hospital workers were in a state of fluid retention, probably because they did not consume sufficient water during the day.

The degree of retention was quantified using a scale, the FRI, which reflects whether the kidneys are currently excreting or retaining fluid. All four components of this FRI scale have previously been used in sports medicine to assess dehydration, which initiates fluid retention. The markers are inter-correlated in a non-linear fashion, but ranges of agreement between them have been published for subjects aged 17–69 years [12]. The individual results of these four markers are displayed in Fig 1 and in Table 2. The argument for constructing a composite index based on these markers is to increase the stability of the assessment [12]. In sports medicine, a urine specific gravity of 1.020 is often used to indicate fluid retention consistent with moderate dehydration [14]. This limit corresponds to a FRI of 4.0 or a body fluid deficit amounting to 3% [12], and was reached by 38% of the cohort studied here. On-call nurses and doctors who present with this degree of fluid retention may exhibit slight cognitive dysfunction [13].

The clinical value of the FRI is not yet clearly established, but a body fluid deficit would appear to occur in patients with a high FRI. The kinetics of infused crystalloid fluid has been reported as the same as in furosemide-induced dehydration in hospital patients with high concentrations of urinary waste products [6,7], and more intravenous fluid was needed to increase the stroke volume of the heart during general anesthesia [8]. High urinary concentrations of waste products have also been associated with higher incidence of postoperative complications [9,10] and higher 30-day mortality in acute geriatric care [11].

The incidence of fluid retention in these cohorts of hospital patients from previous studies was in the same range, or lower, than in the present study. Fluid retention occurred in 13% of patients before gastrointestinal surgery [6], in 15% of geriatric patients [11], and in 31% and 50% of two cohorts before acute hip fracture surgery [9,10]. The incidence was 26% in volunteers before, and 42% after, 90 min of recreational exercise [12]. One widespread belief is that renal conservation of fluid consistent with dehydration is particularly common among hospital patients, but our present results suggest that patients are no more prone to show renal fluid retention than are the hospital workers who care for them.

Men more often retained fluid when compared to women [11,12,14], and the time of the day did not seem to greatly impact the result [14]. Only a minor effect was found for the period of time without fluid intake. The 2-hour period when fluid intake should be avoided before the urine is sampled is believed to provide an FRI score that more closely approximates the steady state situation with respect to body hydration. Many volunteers did not comply with this instruction and, in retrospect, one might even question its importance (Fig 3D). In a previous study, a 2-hour intravenous infusion of 500 ml of crystalloid fluid only reduced the FRI score by less than 1 step [19].

Plasma electrolytes and water-sparing hormones provided little guidance regarding the degree of fluid retention. The renin concentration was higher in men than in women, while plasma levels of sodium, aldosterone, and cortisol did not differ between the subgroups (S2 Table). The difference between volunteers with FRI ≥ 4.0 and the others averaged only 1.5 mosmol/kg (Table 2), which agrees well with recently published data on chronic water depletion by Johnson et al. [20]. Plasma osmolality is otherwise the blood parameter considered to provide the best indication of induced acute water depletion [2,21]. Combined with the lack of any rise in plasma vasopressin, our results suggest that a high FRI in our hospital workers was most likely due to a mixture of mild (sometimes moderate) hypertonic and isotonic dehydration; i.e., the cause was both the insufficient intake of water and the losses of electrolyte-containing fluid [22].

Our finding that an FRI value above 5.0 was invariably associated with albuminuria provides a potential mechanism that could explain isotonic dehydration (Fig 4). A nearly isotonic electrolyte solution given by intravenous infusion was excreted twice as fast in patients with detectable albuminuria than in those with no albuminuria, which suggests that albuminuria promotes excessive excretion of electrolyte-rich fluid [23]. The albuminuria in the present study was modest and usually below the micro-albuminemic range, which is a hallmark of inflammation and worsens the prognosis of diabetes and cardiovascular disease [15,16]. The U-shaped link between albuminuria and a high FRI was an unexpected finding that deserves further study.

Albuminuria occurs due to shedding of the glycocalyx covering the luminal surface of the glomeruli, with inflammation being the most prominent cause. However, albuminuria was not associated with higher urinary concentrations of shedding products, which do not originate only from the glomeruli but arise from the entire cardiovascular system. The concentration of hyaluronic acid followed the FRI value, suggesting a constant rate of excretion. In contrast, the concentration of syndecan-1 was independent of the FRI value. More syndecan-1 was then excreted when the urine flow rate was high, which agrees with previous findings in elderly males [7].

Several previous authors have validated urine sampling for the detection of hypertonic dehydration against exercise-induced reductions of body weight in male athletes and, recently, in volunteers of both genders up to 69 years of age [12]. Urine color and urine specific gravity corresponding to FRI scores of 1–3 represent degrees of normal hydration, scores of 4–5 indicate moderate dehydration, and scores 6 and higher correspond to severe dehydration [3]. A FRI of 5 corresponds to an acute weight loss amounting to about 5%. The urine composition changes very little when dehydration increases from 5% to 7% [2], whereas pre-renal anuria might occur with even more severe dehydration.

Cheuvront et al. found the sensitivity and specificity of urine-specific gravity and urine osmolality to be between 89% and 91% when used to detect induced dehydration in healthy volunteers [21]. Urine color had a sensitivity of only 81%, but a specificity of 97%. Plasma osmolality (301 ± 5 mosmol/kg) had a sensitivity of 90% and specificity of 100%, but the area under the ROC curves was virtually identical for the three urine parameters. However, these data may be dependent on the degree of induced dehydration. Mean values show that urine color, osmolality, and creatinine are better at indicating a dehydration process than is urine osmolality [12]. By contrast, the osmolality seems to change at an earlier stage of the urinary concentration process than do color and creatinine (Fig 1).

Fewer data exist on indices of hydration biomarkers when acute dehydration is not induced by exercise. El-Sharkawy et al. [13] found that 36% of on-call nurses and doctors had a urine osmolality >800 mosmol/kg, which also co-existed with a slight but statistically significant impairment of cognitive function. Johnson et al. [20] compared women who had habitually high and low daily water intakes and also examined how biomarkers change when the fluid intake is modified. They confirmed that urinary color, specific gravity, and osmolality all differed depending on the daily intake of water. The changes in urinary biomarkers that occurred after fluid restriction agreed well with the reduction in body weight that can be predicted from data on exercise [12], while an increase in fluid intake diluted the urinary biomarkers without increasing the body weight. By contrast, the biomarkers derived from blood differed very little depending on the intake of water, which supports our present findings. This study by Johnson et al. also confirms that differences in the urinary biomarkers reported here can be related to the intake of water.

The FRI scale is intended to offer a more robust measure of fluid retention than any one of its four components. Non-linearity between the four markers of fluid retention has been taken into account by identifying ranges of agreement [12]. The problem of occasional misleading measurements was overcome by assuming that several urinary markers are unlikely to be erroneous at the same time. Taking the mean value of four markers scored according to the known inter-correlations existing between them has the benefit of reducing the confounding influences of diet, disease, and medication, which typically change only one of the markers. This composite index is attractive for use in hospital patients where single biomarkers may be affected by factors such as urinary infection (color), vitamins and drugs (color), low muscle mass (creatinine), and variability in the consumption of salt (osmolality) and meat (creatinine).

A method for identifying and eliminating outliers was applied. This refinement procedure assured that at least three of the four scores were consistent with each other. In our hands, urine color was the score that most often conflicted with the others. Urine color depends on subjective judgment, lighting in the room, storage of the color chart, and the quality of the color printer. When setting up the method, urine color should be compared with the other markers and their inter-correlation used for calibration.

The excretion of metals occurs at a fairly stable rate, although inter-individual differences exist. Here, the urinary concentration of the vast majority of the measured metals increased with the FRI value, supporting the likelihood that volunteers having FRI values in the high range belong to a different population than those having FRI in the low range.

Spot urine sampling for measurement of metabolic waste products might not be the ideal way to assess fluid balance [21], but it is a potentially useful tool for assessing the degree of fluid intake in the general population [24]. The analysis is non-invasive and relatively easy to perform. The fluid retention scores show a low week-to-week variability [7] and small hourly changes in the perioperative setting [19]. Limitations include the ability of the subject to provide a urine sample, which should be adequately timed for optimal predictive performance [22,25]. The urinalysis must be interpreted with caution in very sick patients. FRI not only indicates dehydration, but fluid retention of any cause. Some diseases, such as heart failure, have fluid retention as part of the pathophysiology rather than a sign of a body fluid deficit. This issue was not a problem in the present study, which did not include unhealthy people. Moreover, the impaired ability of very old people to concentrate the urine might blur the correlation between serum osmolality and urinary biomarkers of dehydration [26]. However, chronic medication with several drugs might contribute to a raised serum osmolality in the elderly and single urinary biomarkers may be confounded by diet, drugs, and disease.

In conclusion, the results showed great between-subject variability in urinary markers of fluid retention in a cohort of 300 healthy hospital workers. In the absence of physical disease or other possible explanations, we conclude that 38% of the cohort showed renal water conservation consistent with moderate to severe dehydration. Further studies will be needed to evaluate whether these findings have implications for public health.

## Supporting Information

S1 Table Raw data used for the calculations. English Version.
(XLS)

S2 Table Measured parameters depending on gender. (DOCX)

S3 Table Raw data used for the calculations. Original.
(XLS)
