# Supplementary material for: Urinary Analysis of Fluid Retention in the General Population: A Cross-Sectional Study
Source: PLoS One. 2016 Oct 20;11(10):e0164152. doi: 10.1371/journal.pone.0164152 (PMC5072703; doi:10.1371/journal.pone.0164152)
Supplement: S2 Table — (DOCX) [file pone.0164152.s002.docx]

**S2 Table**

Measured parameters depending on gender.

|  | Women | Men | Statistics |
| --- | --- | --- | --- |
|  |  |  |  |
| Demographics |  |  |  |
| N | 199 | 79 |  |
| Age (years) | 43 (13) | 44 (17) | NS |
| Height (cm) | 165 (6) | 180 (6) | P< 0.001 |
| Weight (kg) | 69 (14) | 84 (13) | P< 0.001 |
| Sampling (hour of the day) | 9.7 (3.6) | 9.7 (4.8) | NS |
| Time without liquid (hrs) | 4.4 (3.7) | 5.1 (3.7) | NS |
|  |  |  |  |
| Urine analyses |  |  |  |
| Fluid retention index, FRI | 3.5 (1.2) | 4.1 (1.2) | P< 0.001 |
| Sodium (mmol/L) | 100 (4.9) | 122 (46) | P< 0.001 |
| Potassium (mmol/L) | 59 (32) | 53 (29) | NS |
| Erythrocytes, trace (N, %) | 22 (11%) | 2 (3%) | P< 0.05* |
| pH | 6.1 (0.7) | 6.0 (0.6) | NS |
| Urobilinogen | 3.4 (2.3) | 3.4 (1.4) | NS |
| Nitrite positive | 4 (2%) | 0 | NS* |
| Leucocytes (N, %) | 42 (21%) | 2 (3%) | P< 0.001* |
| Albuminuria (N, %) | 110 (58%) | 44 (56%) | NS* |
|  |  |  |  |
| **Blood analyses** |  |  |  |
| Osmolality (mosmol/kg) | 293.5 (4.2) | 295.3 (3.0) | NS |
| Sodium (mmol/L) | 140.1 (1.9) | 140.0 (1.4) | NS |
| Potassium (mmol/L) | 4.25 (0.32) | 4.06 (0.18) | P< 0.05 |
| Calcium | 2.39 (0.07) | 2.39 (0.08) | NS |
| Cortisol | 372 (153) | 404 (141) | NS |
| Aldosterone | 332 (199) | 295 (171) | NS |
| Renin | 20 (11) | 31 (15) | P< 0.01 |
| Vasopressin | Not detectable | Not detectable | - |
|  |  |  |  |

One-way ANOVA was used for statistics except * contingency table analysis.
